# A Roadmap for the Molecular Farming of Viral Glycoprotein Vaccines: Engineering Glycosylation and Glycosylation-Directed Folding

**DOI:** 10.3389/fpls.2020.609207

**Published:** 2020-12-03

**Authors:** Emmanuel Margolin, Max Crispin, Ann Meyers, Ros Chapman, Edward P. Rybicki

**Affiliations:** ^1^Division of Medical Virology, Department of Pathology, Faculty of Health Sciences, University of Cape Town, Cape Town, South Africa; ^2^Wellcome Trust Centre for Infectious Disease Research in Africa, University of Cape Town, Cape Town, South Africa; ^3^Faculty of Health Sciences, Institute of Infectious Disease and Molecular Medicine, University of Cape Town, Cape Town, South Africa; ^4^Biopharming Research Unit, Department of Molecular and Cell Biology, University of Cape Town, Cape Town, South Africa; ^5^School of Biological Sciences, University of Southampton, Southampton, United Kingdom

**Keywords:** glycosylation, chaperones, calnexin, calreticulin, folding, oligosaccaryltransferase, occupancy, processing

## Abstract

Immunization with recombinant glycoprotein-based vaccines is a promising approach to induce protective immunity against viruses. However, the complex biosynthetic maturation requirements of these glycoproteins typically necessitate their production in mammalian cells to support their folding and post-translational modification. Despite these clear advantages, the incumbent costs and infrastructure requirements with this approach can be prohibitive in developing countries, and the production scales and timelines may prove limiting when applying these production systems to the control of pandemic viral outbreaks. Plant molecular farming of viral glycoproteins has been suggested as a cheap and rapidly scalable alternative production system, with the potential to perform post-translational modifications that are comparable to mammalian cells. Consequently, plant-produced glycoprotein vaccines for seasonal and pandemic influenza have shown promise in clinical trials, and vaccine candidates against the newly emergent severe acute respiratory syndrome coronavirus-2 have entered into late stage preclinical and clinical testing. However, many other viral glycoproteins accumulate poorly in plants, and are not appropriately processed along the secretory pathway due to differences in the host cellular machinery. Furthermore, plant-derived glycoproteins often contain glycoforms that are antigenically distinct from those present on the native virus, and may also be under-glycosylated in some instances. Recent advances in the field have increased the complexity and yields of biologics that can be produced in plants, and have now enabled the expression of many viral glycoproteins which could not previously be produced in plant systems. In contrast to the empirical optimization that predominated during the early years of molecular farming, the next generation of plant-made products are being produced by developing rational, tailor-made approaches to support their production. This has involved the elimination of plant-specific glycoforms and the introduction into plants of elements of the biosynthetic machinery from different expression hosts. These approaches have resulted in the production of mammalian N-linked glycans and the formation of O-glycan moieties *in planta*. More recently, plant molecular engineering approaches have also been applied to improve the glycan occupancy of proteins which are not appropriately glycosylated, and to support the folding and processing of viral glycoproteins where the cellular machinery differs from the usual expression host of the protein. Here we highlight recent achievements and remaining challenges in glycoengineering and the engineering of glycosylation-directed folding pathways in plants, and discuss how these can be applied to produce recombinant viral glycoproteins vaccines.

## Introduction

Since its conception, a major driving force for producing recombinant biologics in plants, or plant molecular farming, has been the potential to cheaply produce pharmaceuticals where they are needed most in the developing world (Rybicki, 2010). Although many of the envisioned advantages of the system still hold true—most notably lower infrastructure requirements and production costs—the capital outlay to build a production facility remains a significant barrier to establishing manufacturing capacity in resource-limited areas (Murad et al., 2019; Margolin et al., 2020a). Additional challenges that hinder the acceptance of plants as a mainstream production platform are low yields for some proteins, and differences in the host biosynthetic machinery which may compromise certain necessary post-translational modifications (PTMs) (Margolin et al., 2020c). Both of these obstacles are probably related: post-translational processing dictates protein folding and misfolded proteins are degraded by quality control systems, therefore accounting for low protein yields in some instances (Strasser, 2018).

Molecular farming has often been proposed as a platform for vaccine and therapeutic manufacturing, particularly in response to pandemic outbreaks (Rigano et al., 2009; Streatfield et al., 2015; Margolin et al., 2020a). This is primarily due to the rapid production time frame, scalability of transient expression and the capacity to mediate most PTMs required for the biological activity of mammalian proteins (Faye et al., 2005; Holtz et al., 2015). Accordingly, many promising biologics have been produced in plant systems to combat human and veterinary pathogens, including multicomponent virus-like particles (VLP) (Thuenemann et al., 2013; Dennis et al., 2018), and numerous recombinant antibodies (Zeitlin et al., 2016; Hurtado et al., 2020; Singh et al., 2020). Objectively, therapeutic antibodies and viral structural components can generally be readily produced in plants and assemble as expected, but the expression of many viral glycoproteins in the system remains an arduous task (Margolin et al., 2018). Advances in expression technologies have improved the yields of many plant-made proteins, and several promising viral glycoprotein vaccines have also now been successfully expressed in recent years—including several from high impact emerging and pandemic viruses (Lomonossoff and D’Aoust, 2016; Margolin et al., 2018). Recent noteworthy examples in the published literature include the Zika virus envelope protein (Yang et al., 2018), the E2 glycoprotein from classical swine fever virus (Laughlin et al., 2019), the HIV envelope glycoprotein (gp140) (Rosenberg et al., 2013; Margolin et al., 2019), the gp350 glycoprotein from Epstein-Barr virus (Margolin et al., 2020b), the Gn glycoprotein from Rift Valley fever virus (Mbewana et al., 2019; Margolin et al., 2020b), the chikungunya E2 glycoprotein (Margolin et al., 2020b) and the hemagglutinin (HA) glycoprotein from both pandemic and seasonal influenza viruses (D’Aoust et al., 2008, 2010; Landry et al., 2010; Mortimer et al., 2012).

Medicago Inc. work in the development of influenza VLP vaccines exemplifies the potential of molecular farming to rapidly respond to pandemic outbreaks. Once established, the platform was used to successfully produce 10 million vaccine doses of hemagglutinin (HA)-based virus-like particles (VLPs) within a month of receiving the sequence (D’Aoust et al., 2010). More importantly, these were protective in stringent preclinical challenge models and showed similar promise in clinical trials (D’Aoust et al., 2008; Landry et al., 2010; Pillet et al., 2015, 2016, 2019). The vaccines constitute a viable alternative to the long-outdated paradigm of egg-based influenza vaccine manufacturing, which has the disadvantages of slow production rates and limited production capacity (D’Aoust et al., 2010). Notably, the vaccines comprise enveloped VLPs which are probably more potent immunogens than other non-particulate subunit vaccines under development (D’Aoust et al., 2008). More recently, following the emergence of SARS-CoV-2 and the ensuing global pandemic, iBio Inc., Medicago Inc., and Kentucky Bioprocessing Inc., have all confirmed the production of candidate vaccines in *Nicotiana benthamiana* (iBio, 2020; Medicago, 2020; Ziady, 2020). The publicly-available details of these vaccines are currently limited, but Medicago’s VLP-based vaccine has already initiated a Phase I clinical trial [NCT04450004] (Kentucky, 2020).

In contrast, attempts to express other more complex viral glycoproteins in plants have often been less successful (Margolin et al., 2018). In many cases, poor protein accumulation has been associated with leaf necrosis soon after expression of the target protein. This phenotype indicates severe endoplasmic reticulum stress in response to the accumulation of misfolded proteins, and may suggest a fundamental incompatibility with the host folding machinery (Hamorsky et al., 2015). Given the complex maturation of viral glycoproteins along the secretory pathway, and their reliance on these processing events to co-ordinate their folding, this may not be surprising (Watanabe et al., 2019). Host-derived glycosylation is central to glycoprotein maturation and trafficking, and the extensive glycosylation of many viral glycoproteins probably exceeds anything that would naturally be produced in a plant system (Watanabe et al., 2019). It would also be naïve to discount the influence of the plant glycosylation machinery—compared to the mammalian hosts of these viruses—as plants differ with respect to N-glycan sequon occupancy, glycan processing and do not naturally support mammalian-type O-glycan biosynthesis.

Whilst viral glycoprotein production in plants certainly faces significant challenges, recent advances in molecular engineering have prompted the development of new approaches to humanize plant glycosylation, and to accommodate the maturation of proteins which would not otherwise occur *in planta* (Margolin et al., 2020c). These strategies provide new hope for the development of vaccines and other biologics, production of which would not previously have been possible. In this article we highlight these advances in the molecular engineering of glycosylation and glycosylation-directed folding in plants, and discuss how they can be implemented to produce well-folded and appropriately glycosylated recombinant viral glycoproteins for use as vaccines.

## Pit Stop 1: Glycosylation of Virion-Associated Viral Glycoproteins

The majority of enveloped viruses that impact human health display virally-encoded glycoproteins on the virion surface (Bagdonaite and Wandall, 2018; Watanabe et al., 2019). These proteins are responsible for mediating fusion with the host cell, and therefore determine the host range and infectivity of the virus (Rey and Lok, 2018). A common feature of envelope fusion glycoproteins is the presence of host-derived glycans which are central to the virus lifecycle (Watanabe et al., 2019). There are multiple selective pressures shaping both the frequency and distribution of these glycans, with different factors influencing N- and O-linked glycosylation. Both classes of glycosylation can enhance the physicochemical stability of the glycoprotein, but in addition, N-linked glycans facilitate protein folding through their role in the calnexin/calreticulin chaperone-mediated folding cycle. The complexity of viral glycoprotein structures, such as the use of extensive disulfide bonding, often leads to dependence on glycan-mediated folding pathways. The dependence of viral glycoproteins on these folding mechanisms, compared to typical mammalian host glycoproteins, is demonstrated by the antiviral activity of small-molecule inhibitors that block glycan-mediated chaperone interactions (Mehta et al., 1998; Chang et al., 2013; Tyrrell et al., 2017; DeWald et al., 2020). The interconnection between glycosylation and the formation of the target disulfide bond network in turn is illustrated by the recruitment of oxidoreductases during protein folding (Molinari and Helenius, 1999), and the presence of thioredoxin-like domains within the UDP-glucose:glycoprotein glucosyltransferase folding sensor (Roversi et al., 2017). The addition of artificial disulfide bonds in recombinant viral glycoproteins is also emerging as a key strategy to stabilize recombinant viral glycoprotein mimetics to ensure that they reproduce the native glycoprotein architecture (Binley et al., 2000; Sanders and Moore, 2017; Allen et al., 2018).

The dependence on N-linked glycans for chaperone-mediated folding events has probably also facilitated the structural incorporation of some glycans into the protein fold. In HIV-1, for example, the glycan at Asn262 of the envelope glycoprotein forms extensive contacts with the protein surface, and its absence leads to an almost complete loss of protein fold integrity and viral infectivity (Huang et al., 2012; Kong et al., 2015). Similarly, glycans shield and stabilize the protein in many other viruses such as SARS-CoV-2 (Zhao et al., 2020), Lassa (Watanabe et al., 2018), and influenza (Hebert et al., 1997). While not involved in chaperone-mediated folding, O-linked glycosylation is fundamental to the structure of mucin-like domains (Gerken et al., 1989): these are present in proteins of many viral families, such as the *Filoviridae* (Tran et al., 2014) and *Herpesviridae* (Norden et al., 2015). The presence of O-linked glycans outside extensive mucin-like regions can also be detected in some cases, and plays a similarly important role in viral fitness (Silver et al., 2020).

In addition to their role in glycoprotein folding and assembly, glycans can have numerous influences on viral pathobiology. One well-documented selective pressure for the addition of glycosylation sites is immune evasion. In the extreme, viruses such as HIV-1 which persist within an infected individual by evolving away from the specificities of the host’s adaptive immune response, have very high densities of envelope protein glycosylation (Wei et al., 2003). In another setting, hit-and-run viruses such as influenza can accumulate and redistribute glycans seasonally as part of the antigenic drift facilitating continued circulation within the population (Wu and Wilson, 2017).

Viral glycans can also have functions within the host, and it can often be difficult to understand if these properties have been directly selected for, or if they have arisen as a consequence of immunological selective pressures. Many viral glycoproteins contain complex N-glycans that are decorated with galactose, fucose and sialic acid (Bowden et al., 2008; Collar et al., 2017; Watanabe et al., 2020a, b). In contrast, however, the density of N-linked glycans on HIV virions is sufficiently high that the glycan network acts as a steric blockade to the enzymes that mature glycans from oligomannose-type to complex-type (Behrens and Crispin, 2017). The resulting “mannose patch” facilitates lectin-mediated viral trafficking but also lectin-mediated complement activation (Borggren and Jansson, 2015; Mason and Tarr, 2015). Given the high density of viral glycans, it could be envisioned that these could be targeted by antibodies. However, a significant hurdle in the antibody-mediated recognition of viral glycans is that many glycan-binding B-cells are autoreactive, and are therefore eliminated by central tolerance (Haynes and Verkoczy, 2014). A couple of exceptions to this general rule are the antibody-mediated barrier to viral transmission arising from blood group incompatibility (Neil et al., 2005), and in zoonosis where non-human glycan epitopes can be displayed on the initially infecting virions (Crispin et al., 2014). Despite the issue of tolerance, many individuals infected with HIV-1 go on to develop broadly neutralizing antibodies where regions of glycans form part of the epitope (Crispin et al., 2018). While the antigenic diversity means that the individuals still require antiviral drugs to control the infection, these broadly neutralizing antibodies show promising protection in viral challenge models and their elicitation is a major goal in vaccine design programs (Sok and Burton, 2018; Haynes et al., 2019). It is also noteworthy that antibodies targeting glycans have also been identified in other viral systems, such as against coronaviruses (Pinto et al., 2020).

Therefore, it is often important that recombinant immunogens are capable of reproducing features of native viral glycosylation (Behrens et al., 2017; Krumm and Doores, 2020). Monitoring glycosylation is consequently increasingly performed when manufacturing viral glycoproteins for clinical trials (Dey et al., 2018). Another influence of glycosylation in the setting of viral immunogens is the impact of glycosylation on immunogen trafficking and the resulting immune response (Tokatlian et al., 2019). It is therefore also important to consider engineering of immunogen glycosylation in the optimization of vaccine candidates.

## Pit Stop 2: Implications of Plant-Specific N-Glycans for Viral Glycoprotein Vaccine Development

It is well-established that the expression of heterologous proteins in plants yields N-glycans that are distinct from those present on mammalian glycoproteins (Strasser, 2016). The most well-documented difference is the presence of plant-specific complex glycans that contain β1,2-xylose and α1,3-fucose (Strasser, 2016). Truncated (paucimannosidic) and elongated (Lewis A epitope) glycoforms are also observed on plant-produced proteins, but are comparatively less abundant and have not been studied as extensively as complex glycans (Montero-Morales and Steinkellner, 2018). Paucimannosidic glycans arise from the enzymatic removal of N-acetylglucosamine (GlcNAc) from the glycan core, and are present on vacuolar and some extracellular glycoproteins (Liebminger et al., 2011). In contrast, Lewis A structures arise from the extension of GlcNAc structures with β1,3-galactose and α1,4-fucose (Fitchette-Laine et al., 1997). Lastly, plants also lack the biosynthetic machinery for sialic acid synthesis, and consequently unlike those in mammalian cells, plant-produced glycans are not sialylated (Zeleny et al., 2006).

Whilst there has been a limited number of reports describing the glycosylation of plant-produced viral glycoproteins, those that have been published are consistent with observations for other plant-produced proteins. The prototypical influenza VLP vaccine produced by Medicago Inc., for example, contains typical complex plant glycan structures, as well as some paucimannosidic and Lewis A-type glycans, on the HA glycoprotein (Ward et al., 2014). Similarly, a truncated HIV envelope gp140 that was produced in *N. benthamiana* contained complex plant-specific glycans and oligomannose glycans, as expected (Rosenberg et al., 2013). The biggest concern with these plant-specific glycans was that they would be recognized as foreign by the human immune system, and that this could either result in a hypersensitive response or rapid clearance following immunization which would diminish their efficacy (Bosch and Schots, 2010). It has now been established that plant-derived glycoforms are safe in humans, and that they do not impair the immunogenicity of plant-produced influenza VLPs containing the viral glycoprotein (Ward et al., 2014). These vaccines were reported to be safe even in volunteers with pre-existing plant allergies, and although some transient IgG and IgE responses were observed to glycoepitopes they did not elicit any adverse effects (Ward et al., 2014). This landmark study addresses long-standing safety concerns that potentially undermined the use of plant-made proteins for human use. Furthermore, these observations also confirm the appropriate trafficking of complex viral glycoproteins through the secretory pathway, which is a critical determinant for post-translational modifications. However, it remains to be determined if repeated immunization—as would be expected in the case of an annual influenza vaccine—leads to unwanted responses against plant-derived glycans. A similar concern exists for other vaccines that require booster immunizations. Similarly, it is unclear if immune responses toward glycoepitopes would be exacerbated following immunization with more heavily glycosylated viral glycoproteins—such as in the case of plant-produced SARS-CoV-2 spike-based vaccines (Watanabe et al., 2020a). Anti-drug responses are highly undesirable and have been associated with reduced clinical efficacy of other pharmaceuticals, or even anaphylaxis in some cases (Arnold and Misbah, 2008; Hsu and Armstrong, 2013; Mok et al., 2013). The elicitation of anaphylactic reactions is likely related to the abundance of the foreign epitope, and some human-approved antibodies contain low levels of immunogenic glycoforms, albeit at lower levels than typical plant glycoforms (Beck and Reichert, 2012). Recently, several studies have suggested that some proteins may be under-glycosylated (lower site occupation) when expressed in plants (Castilho et al., 2018). Glycan “holes” arising from partial sequon occupancy are a common artifact of recombinant expression systems (Cao et al., 2018; Struwe et al., 2018), and are generally undesirable for vaccine immunogens as they may lead to distracting non-neutralizing antibody responses (Derking et al., 2020). To date this has only been reported for a small number of plant-produced proteins—mainly antibodies and some enzymes—and has not yet been adequately explored for viral glycoproteins (Zeitlin et al., 2016; Castilho et al., 2018). However, this phenomenon may account for the difficulties in producing certain viral glycoproteins in plants (Margolin et al., 2018). The loss of even a single glycan can compromise glycoprotein trafficking along the secretory pathway in natural infection, preventing proper processing or export to the cell surface (Moll et al., 2004; Shi and Elliott, 2004; Luo et al., 2015; Shen et al., 2016). A similar reliance on glycosylation can also be expected for the production of these proteins in heterologous expression systems. Given the role of glycans in directing glycoprotein folding, lower levels of glycan occupancy could be expected to compromise chaperone-mediated folding and to result in increased aggregation or impaired oligomerization. The latter was recently illustrated for recombinant IgA produced in *N. benthamiana*, where under-glycosylation in the heavy chain tail piece resulted in inefficient dimerization (Göritzer et al., 2020).

**FIGURE 1:**
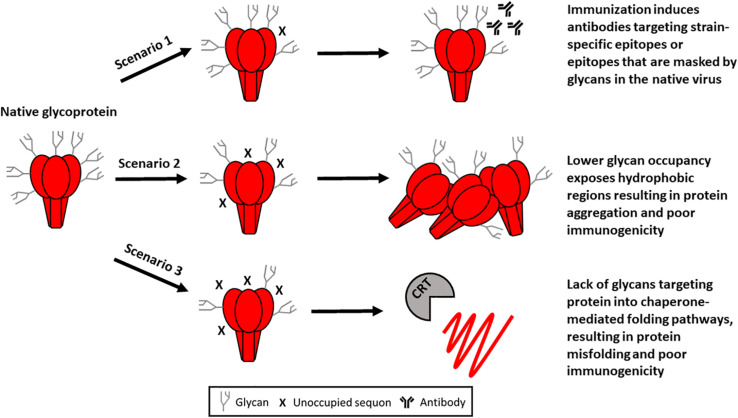
Potential impact of under glycosylation on the immunogenicity of a plant-produced viral glycoprotein. In scenario 1 immunization results in the induction of antibodies which target an epitope on the recombinant protein that is obscured by a glycan in the wildtype virus. Therefore, the antibodies induced are unable to neutralize the virus as this epitope is masked. Alternately, antibodies may target strain-specific holes in the glycan shield distracting the immune response from epitopes that are targets of broadly neutralizing antibodies. In scenario 2, lower glycan occupation exposes hydrophobic stretches of the protein which are usually shielded by glycans. This results in protein aggregation which occludes important epitopes of the protein for immunization. In scenario 3, poor glycan occupancy precludes recognition by ER-resident chaperones which mediate glycoprotein folding. This results in misfolded protein which may elicit a high magnitude immune response that is not protective (CRT, calreticulin).

In the context of a recombinant viral glycoprotein, under-glycosylation would be expected to negatively impact the immunogenicity of a vaccine antigen in several ways (Figure 1). This could explain the lack of neutralizing antibodies in rabbits immunized with a plant-produced HIV gp140 envelope trimer, despite the presence of high levels of binding antibodies (Margolin et al., 2019). Under-glycosylation could also potentially account for the protein aggregation that has been reported following the transient expression of the HIV envelope glycoprotein in plants (Rosenberg et al., 2013; Margolin et al., 2019).

Although it remains to be experimentally determined for viral glycoproteins, paucimannosidic structures are likely to be enriched in proteins which naturally traffic to the plasma membrane, which is the site of β-N-acetylhexosaminidase 3 (HEXO3) in plants (Liebminger et al., 2011; Shin et al., 2017). This has been documented for other plant produced proteins such as human α1-antitrypsin (Castilho et al., 2014) and bovine follicle stimulating hormone (Dirnberger et al., 2001), where these glycan processing events increased heterogeneity of the recombinant proteins and impacted their biological activity. Paucimannosidic glycans do not naturally occur on viral glycoproteins derived from humans, although they are common in viruses from insect vectors (Crispin et al., 2014). It is therefore interesting to consider for production of vaccines against zoonotic arboviruses that plant expression systems may capture features of insect-derived viruses. Exposure of terminal mannose residues following processing may accelerate protein turnover following recognition by lectin receptors (Yang et al., 2015), and this may prevent sustained antigenic stimulation which is important for the induction of an appropriate immune response. On the other hand, mannose-terminating glycans may also aid in trafficking to follicular dendritic cells (Tokatlian et al., 2019) and presentation of glycan-based epitopes shared with those of insect-derived viruses. The Lewis A epitope is the least abundant glycan species in plant-produced proteins but has been described for certain heterologous proteins, including Medicago’s influenza hemagglutinin-based VLP vaccines (Le Mauff et al., 2015). Given the paucity of glycosylation data available for plant-produced viral glycoproteins, it is difficult to establish how common this modification is. It is also difficult to predict its influence for vaccination.

The absence of mammalian-like O-glycan machinery in plants may also pose challenges to the production of viral glycoproteins containing mucin-like domains. Similarly, the absence of mammalian sialic acid residues is a further complication and may be undesirable in some settings. For example, sialic acid has been determined to form part of the epitope of some broadly neutralizing antibodies against HIV (Pancera et al., 2013). However, in the context of Medicago’s influenza VLP vaccines, the absence of sialic acid is highly advantageous as this enables the budding of the viral hemagglutinin as particles in the absence of other accessory proteins (D’Aoust et al., 2008). Sialic acid would otherwise tether the glycoprotein to the host cell, necessitating the expression of neuraminidase to sever this linkage for budding to occur (Chen et al., 2007).

## Destination 1: Approaches to Produce Viral Glycoproteins in Plants With “Native-Like” Glycosylation

Long-standing concerns about the potential impact of plant-specific glycoforms has prompted extensive efforts to humanize the plant glycosylation machinery (Montero-Morales and Steinkellner, 2018). These approaches have successfully yielded human-like glycoforms, by implementing tailored approaches to generate specific glycan moieties (Margolin et al., 2020c). Given that viral glycoproteins are amongst the most extensively glycosylated pharmaceutical targets, it would seem promising to apply such approaches to this class of protein.

**FIGURE 2:**
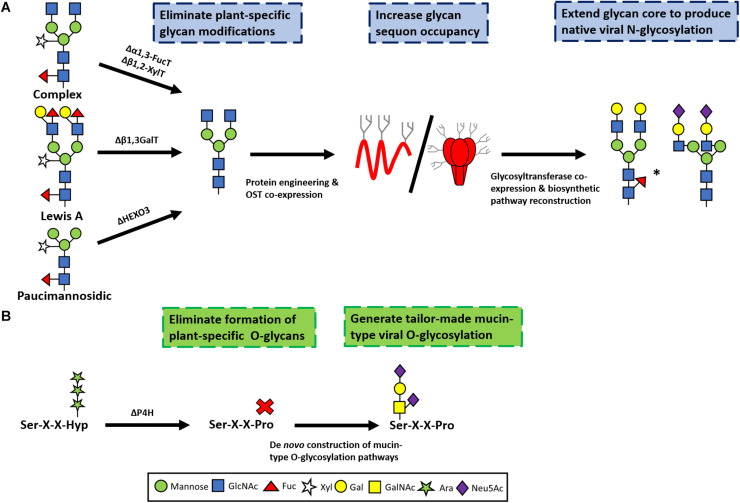
Glycoengineering approaches to produce authentic viral N-glycan **(A)** and O-glycan **(B)** structures in plants. In **(A)** typical plant-derived complex, Lewis A and paucimannosidic glycans are depicted. In order to produce authentic viral-type glycosylation plant-specific glycan processing events need to be eliminated. This involves knocking out the activity of α1,3-fucosyltransferase (Δα1,3-FucT) and β1,2-xylosyltransferase (Δβ1,2-XylT) to prevent the formation of plant-specific complex glycans. Similarly, mitigating the activities of β1,3-galactosyltransferase (GalT) will prevent the formation of Lewis A glycans. Lastly, suppression of β-hexosaminidase 3 (ΔHEXO3) will avoid processing to yield paucimannosic structures. These approaches may need to be combined with strategies to improve the glycan occupancy, such as by engineering the host sequence or by expressing heterologous oligosaccaryltransferases. The resulting glycan core can then serve as a substrate to generate tailor-made glycoforms. The co-expression of the necessary glycosyltransferases will support the formation of native viral extensions; including α1,6-fucosylation, β1,4-galactosylation and α-2,6-sialylation. The efficiency of the glycosyltransferases may be variable *in planta* and could result in partial occupancy (indicated by ^∗^). In **(B)** the elimination of prolyl 4-hydroxylases (ΔP4H) will prevent the undesired conversion of proline to hydroxyproline. Subsequently, the biosynthetic machinery required for mucin-type glycosylation can be expressed *in planta* to yield viral glycoforms with typical extensions.

The first step toward humanizing N-glycosylation in plants was achieved by eliminating the enzymes responsible for imparting plant-specific complex glycans (β1,2-xylosyltransferase and α1,3-fucosyltransferase). This was originally achieved using RNA interference to down-regulate expression of the target genes (Strasser et al., 2008), but more recently the CRISPR/Cas9 system was used to completely ablate activity of the enzymes (Jansing et al., 2019). RNA interference has also been applied to prevent the formation of paucimannosidic glycans by mitigating β-hexosaminidase activity in *N. benthamiana* (Shin et al., 2017), and the targeted knockout α1,4-fucosyltransferases and β1,3-galactosyltransferases in cultured moss cells successfully eliminated the formation of Lewis A structures (Parsons et al., 2012). These approaches potentially allow for the production of viral glycoproteins lacking undesirable glycan modifications *in planta*, essentially yielding a core structure that can be modified to generate tailored N-glycans with mammalian-type extensions (Figure 2).

In combination with these approaches, the expression of heterologous glycosyltransferases can generate authentic mammalian-like N-glycosylation. Notable achievements in this regard have included the production of glycans with β1,4-galactose (Schneider et al., 2015; Stelter et al., 2020) and sialic acid extensions (Castilho et al., 2010; Kallolimath et al., 2016), as well as the formation of bisected, branched and multiantennary structures (Castilho et al., 2011; Nagels et al., 2011). These achievements establish an important precedent for reproducing mammalian-type N-glycosylation in plants, and constitute a useful framework for production of authentically-glycosylated viral glycoproteins.

The recent observation that some proteins may be under-glycosylated in plants raises concerns that this may be an important constraint for the production of heavily glycosylated biologics in the system (Jarczowski et al., 2016; Castilho et al., 2018; Montero-Morales et al., 2019; Göritzer et al., 2020; Margolin et al., 2020c; Singh et al., 2020). It is presently unclear how widespread this phenomenon is, particularly in the context of viral glycoproteins, as few published reports have described the quantitative glycosylation analysis of plant-produced proteins. Glycan occupancy is dependent on the host oligosaccaryltransferase (OST) complex which transfers the preassembled glycan precursor (Glc_3_Man_9_GlcNAc_2_) to the N-X-S/T sequon of the protein (where X is any amino acid except proline) (Mohorko et al., 2011). In human cells, the OST complex exists in two different forms which are defined by the presence of either the STT3A or STT3B catalytic subunits (Ramirez et al., 2019). In addition to the catalytic subunits, each complex also contains a shared core of non-catalytic components as well as isoform-specific subunits (Ramirez et al., 2019). The two complexes have complimentary functions in directing protein glycosylation: STT3A mediates co-translation glycosylation whereas STT3B is responsible for post-translational glycosylation (Ruiz-Canada et al., 2009). The composition of the OST complex in plant cells has not been as well described, but homologs of the human catalytic subunits have been reported for *Arabidopsis thaliana* (Koiwa et al., 2003; Strasser, 2016).

Although the efficiency of glycosylation is influenced by a number of variables—including glucose availability (Liu et al., 2014), the proximity of adjacent sequons (Shrimal and Gilmore, 2013), amino acid sequence of the glycan sequon (Gavel and von Heijne, 1990) and flanking regions (Murray et al., 2015; Huang et al., 2017)—under glycosylation *in planta* has mainly been attributed to the unique recognition preferences of the plant OST complex (Margolin et al., 2020c). Accordingly, the co-expression of the *Leishmania major* LmSTT3D OST enzyme has been reported to improve the glycan occupancy of a range of substrates in plants (Castilho et al., 2018), and it has been proposed that the co-expression of other single subunit OSTs may confer a similar benefit (Margolin et al., 2020c). This has yet to be explored for any plant-produced viral glycoprotein but may prove to be an important approach to ensure adequate glycosylation of complex glycoprotein antigens in the system. Targeted sequence changes may also prove useful to improve glycosylation, including modification of the glycan sequon (Gavel and von Heijne, 1990) or the proximal regions (Murray et al., 2015; Jarczowski et al., 2016; Huang et al., 2017).

The second major type of glycosylation relevant to producing viral glycoprotein vaccines in plants is mucin-type O-glycosylation. In mammalian cells this arises from the addition of N-acetylgalactosamine (GalNAc) to serine, threonine and tyrosine residues in the protein, which are then extended with various monosaccharides (Halim et al., 2011). Consequently, O-glycosylation can yield highly variable structures (Watanabe et al., 2019). Plants do not naturally support the synthesis of mammalian-type O glycans, but instead often convert proline residues to hydroxyprolines, which are then extended with arabinose (Karnoup et al., 2005; Pinkhasov et al., 2011). Similar to the challenge of humanizing N-glycosylation, the production of viral glycoproteins with authentic O-glycosylation may need to consider both the elimination of undesirable plant-specific modifications and mammalian O-glycan extensions. Theoretically, in order to avoid plant-specific O-glycan modifications, the activities of the responsible prolyl 4-hydroxylase enzymes needs to be suppressed (Moriguchi et al., 2011). However, it is unclear if these modifications are even present on plant-produced viral glycoproteins; and data describing their site-specific glycosylation is notably lacking. Furthermore, in some cases it may even be beneficial to engineer the glycoprotein to remove mucin-like regions as they can be highly variable and may obscure vulnerable epitopes (Fusco et al., 2015; Rutten et al., 2020).

Nonetheless, producing viral glycoproteins in plants with native O-glycans will require the entire biosynthetic pathway to be expressed *de novo*—as has been the case for other non-viral targets. Encouragingly, mucin-type glycosylation has already been successfully achieved with several model proteins in plants, by introducing the cellular machinery required to mediate the transfer and elongation of GalNAc (Daskalova et al., 2010; Yang et al., 2012; Dicker et al., 2016). This even includes sialylated mucin-type O glycans where the biosynthetic machinery for both O-glycosylation and sialylation were introduced into the plant expression host (Castilho et al., 2012). These reports demonstrate the flexibility of plant expression platforms which support the co-expression of multiple components of the cellular machinery from different hosts, or even, in the extreme, of entire biosynthetic pathways. This is highly encouraging as, realistically, in order to produce authentic viral glycoproteins from certain viruses both N-and O-glycan engineering approaches will probably need to be combined. The obvious drawback, however, is that a pre-existing knowledge of the native viral glycosylation is required to implement these approaches, and therefore if glycoengineering is necessary to produce a target antigen, this may preclude the use of the platform to rapidly respond adequately to a pandemic outbreak.

## Destination 2: Glycosylation-Directed Folding of Viral Glycoproteins

**FIGURE 3:**
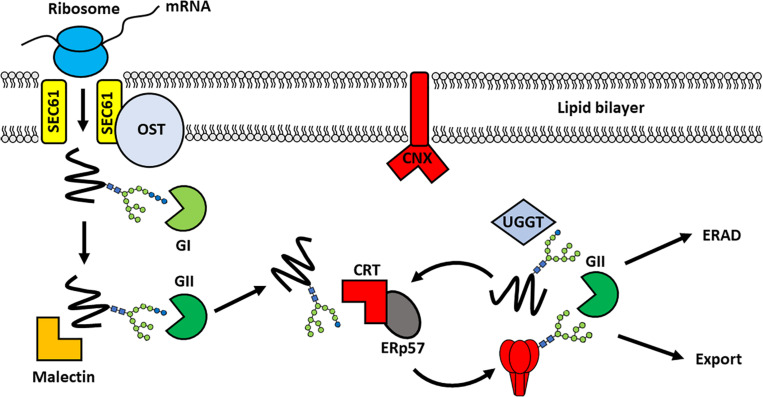
Chaperone-mediated folding of viral glycoproteins in the ER. Following translation, the nascent protein enters into the ER through the translocon pore (SEC61) and is glycosylated by the membrane-bound oligosaccaryltransferase complex. Processing of the glycan by α-glucosidase I (GI), to remove the outermost glucose residue, enables recognition by Malectin. The removal of a second glucose yields a monoglucosylated structure that is the substrate for the lectin binding chaperones: calnexin (CNX) and calreticulin (CRT). These chaperones recruit other folding partners, such as the oxidoreductase ERp57, to support glycoprotein folding. Once the glycoprotein is appropriately folded the final glucose is removed by GII to release the protein from the CNX/CRT folding cycle. The glycoprotein can then traffic into the Golgi apparatus for further modifications, including proteolytic cleavage and glycan maturation. In contrast, aberrantly-folded glycoproteins are reglucosylated by UDP:glucose glycoprotein glycosyltransferase (UGGT) causing their retention in the CNX/CRT folding pathway for another round of chaperone-mediated folding. Terminally misfolded proteins are eventually targeted for Endoplasmic reticulum-associated degradation (ERAD) to prevent misfolded proteins progressing through the secretory pathway. A single glycan is depicted for simplicity.

The endoplasmic reticulum (ER) is the main site of viral glycoprotein folding and quality control. Following translation, the nascent protein is glycosylated by the host OST complex and trafficks into the ER for chaperone-mediated folding, disulfide bond formation and oligomerization. The folding process is carefully regulated by host-derived glycans which co-ordinate chaperone-mediated folding and impose quality control checkpoints, to ensure that only correctly folded protein progresses into the Golgi apparatus for further maturation. Whilst non-glycosylated proteins are targeted into the classical chaperone folding pathway, glycoprotein folding is coordinated by the lectin chaperones calnexin (CNX) and calreticulin (CRT) (Figure 3; McCaffrey and Braakman, 2016). The choice between the two pathways appears to be dictated by proximity of glycans to the amino-terminus of the protein, although both pathways can act cooperatively (Molinari and Helenius, 2000). The classical chaperone folding pathway comprises of members of the heatshock protein family, such as Binding-immunoglobulin protein (BiP), which bind to hydrophobic stretches of protein to assist with folding and to prevent protein aggregation (Adams et al., 2019). In contrast, both CNX and CRT recognize glycans directly (Hammond et al., 1994). Although their substrate recognition is essentially the same, CNX is associated with the ER membrane whereas CRT is soluble (Wada et al., 1995). Accordingly, CNX generally preferentially associates with transmembrane glycoproteins. CRT in contrast, typically participates in the folding of soluble glycoproteins, although it is noted that the proximity of glycans to the membrane may also influence the choice of the 2 chaperones (Hebert et al., 1997).

Glycan processing co-ordinates the sequential interaction of the glycoprotein with different folding partners in the CNX/CRT folding cycle. The removal of the first glucose by α-glucosidase I enables recognition of the di-glucosylated glycan (Glc_2_Man_9_GlcNAc_2_) by malectin, which acts in concert with ribophorin I to prevent the secretion of misfolded proteins (Schallus et al., 2008; Chen et al., 2011; Takeda et al., 2014). This is followed by the removal of a second glucose by α-glucosidase-II, resulting in recognition of the monoglucosylated sugar (GlcMan_9_GlcNAc_2_) by CNX and CRT (Hammond et al., 1994). The association of the glycoprotein with CNX/CRT promotes folding and disulfide bond formation through interaction with various foldases (Schrag et al., 2003). These include oxidoreductases such as protein disulfide isomerase and ERp57, and peptidylproline isomerases, such as cyclophilin B (Molinari and Helenius, 1999; Oliver et al., 1999; Kozlov et al., 2010). Removal of the final glucose by α-glucosidase-II releases the glycoprotein from the CNX/CRT folding cycle allowing the protein to continue its progress along the secretory pathway (Hebert et al., 1995). Misfolded glycoproteins, however, are reglucosylated by UDP-glucose: glycoprotein glycosyltransferase (UGGT) targeting them back into the CNX/CRT pathway for another round of chaperone-mediated folding (Sousa and Parodi, 1995; Ritter and Helenius, 2000). Proteins that are unable to assume their correct conformations are eventually targeted for ER-associated degradation (ERAD). Aberrantly folded proteins are distinguished from well-folded proteins by sequential mannose trimming, conducted by ER α-mannosidase I (ERManI) and ER-degrading α-enhancing-mannosidase-like proteins (EDEM 1–3) (Caramelo and Parodi, 2015).

Whilst these carbohydrate-driven folding and quality control pathways are present in plants, it is unclear how their divergence from mammals could impact the folding of complex viral glycoproteins. The observation that low viral glycoprotein accumulation in plants is often associated with tissue necrosis suggests that the plant cellular machinery may not always support the efficient folding of these proteins. It was therefore proposed that the endogenous plant chaperone machinery was incompatible with the folding of certain viral glycoproteins (Margolin et al., 2018). Although this may be true to some extent, it is also overly simplistic as viral glycoprotein folding is also reliant on early events in the glycosylation pathway (preceding chaperone-mediated folding), and proteolytic processing which occurs later in the Golgi apparatus. Unsurprisingly, *in silico* analyses have highlighted considerable sequence divergence of plant homologs from human chaperones that are known to mediate glycoprotein folding (Margolin et al., 2020b).

Based on this observation, the expression of several human chaperones was explored to improve the production of a soluble HIV envelope gp140 antigen in *N. benthamiana*. Early attempts to express the protein resulted in low yields and the purified antigen was prone to forming aggregates which were poorly resolved by SDS-PAGE (Margolin et al., 2019). Following the co-expression of human CRT, an approximately 13-fold increase in relative expression of the antigen was observed, although the levels of unresolved protein aggregates also appeared to increase proportionately (Margolin et al., 2020b). Encouragingly, following co-expression of the chaperone, both the necrotic phenotype and representative markers of ER-stress were reduced (Margolin et al., 2020b). Further work is still needed to determine the impact of the co-expressed chaperone on protein antigenicity—based on reactivity with human-derived monoclonal antibodies—and immunogenicity in vaccinated animals. A similar increase in protein accumulation was also observed when CRT was co-expressed with other soluble viral glycoproteins. In several instances this enabled the production of glycoproteins which could not be expressed at detectable levels in the absence of the co-expressed chaperone. These include glycoproteins from Rift Valley fever virus, chikungunya virus and Epstein-Barr virus (Meyers et al., 2018; Margolin et al., 2020b). This approach has also been applied to producing recombinant antibodies, although the impact was more modest (Meyers et al., 2018; Göritzer et al., 2020).

Lastly, the co-expression of human chaperone proteins has also been combined with furin expression to accommodate glycoprotein processing in *N. benthamiana*, and this could similarly be applied to other proteases that are required for viral glycoprotein maturation if they do not occur in plants, or if the endogenous levels are too low to exert the desired effect (Figure 2B; Margolin et al., 2020b). An alternate approach to co-expressing furin, is to replace the cleavage site with a flexible linker, which has been shown to promote the assembly of native-like HIV envelope trimers in mammalian production systems (Georgiev et al., 2015; Sharma et al., 2015; Sarkar et al., 2018). This approach has recently been explored to producing cleavage-independent viral glycoproteins in plants, but further work remains to determine how closely they resemble their mammalian cell-produced counterparts (Margolin et al., 2019, 2020b).

## Conclusion

The advanced progress toward licensure of Medicago’s seasonal influenza vaccine and recent progress in the production of plant-produced SARS-CoV-2 vaccine candidates has resulted in growing recent interest in plant molecular farming of viral glycoproteins (Medicago, 2019). These examples represent pivotal landmarks in the field that highlight the potential of the platform for rapid large-scale production and translation into clinical development. Importantly, they confirm that, despite the differences in the plant cellular machinery compared to mammalian cells, efficacious vaccines can be produced in the system—and usually at a lower cost of materials. In addition to these developments, various host engineering approaches have also been conceived to accommodate certain PTMs, and other processing events, which would not otherwise occur appropriately in plants (Margolin et al., 2020c). These have enabled the successful production and processing of viral glycoproteins which previously could not be produced in plants (Margolin et al., 2020b). However, the production of many other complex viral glycoproteins in plants remains a challenge, and may require further humanization of the biosynthetic machinery to produce feasible vaccine immunogens. Addressing under-glycosylation and different glycan processing events will probably constitute a critical component of this endeavor. Fortunately, many of these approaches have already been developed for other biopharmaceutical products, and could easily be applied to these targets (Margolin et al., 2020c).

In conclusion, the molecular farming of viral glycoproteins is gathering momentum, and the integration of glycoengineering and other host engineering approaches will be an important focus in addressing the production of next-generation glycoprotein vaccines where they are most needed.

## Author Contributions

EM and MC led the writing of the manuscript. RC contributed to the molecular biology aspects of the article. AM and ER contributed to the biopharming aspects of the article. EM drafted the figures with input from the other contributing authors. All authors made a significant contribution to the manuscript and approved the final article for submission.

## Conflict of Interest

EM, AM, and ER have filed a patent describing the co-expression of chaperone proteins to improve the production of heterologous polypeptides in plants. The remaning authors declare that the research was conducted in the absence of any commercial or financial relationships that could be construed as a potential conflict of interest.
